# Project *Muskan*: Social responsibility of the plastic surgeon

**DOI:** 10.4103/0970-0358.44920

**Published:** 2008

**Authors:** Yogesh C. Bhatt, Nikhil S. Panse, Kinnari A. Vyas, Harpreet S. Bakshi, Mangesh S. Tandale, Rajat K. Shrivastav

**Affiliations:** Department of Plastic Surgery, SSG Hospital and Medical College, Baroda, India

**Keywords:** Dairy, government, muskan, nongovernment organization

## Abstract

Although exact statistics are not available, Indian plastic surgeons see around 7,00,000–8,00,000 burn admissions annually with around 10,00,000 cleft patients yet to be operated. In spite of this voluminous load, India does not have national health programs for the various deformities Indian plastic surgeons typically treat. As Plastic Surgeons, it is our social responsibility to treat these patients and bring ‘*muskan*’ (smile in Hindi) back into their lives. Project Muskan was initiated as an innovative model for targeting these patients and is probably one of its kind in the field of plastic surgery in our country. It is unique because it is a perfect collaboration of government institutions, a Non Government Organization (NGO), and cooperative sectors providing free health care at the doorstep. Identification of the patients was done with the help of the extensive milk dairy network in the state of Gujarat. Provision of transport and other facilities was done by the NGOs and quality health care provision was taken care of by the government hospital. Project Muskan started from a single village but now covers around 3000 villages and tribal areas of Gujarat. It is a system that can be easily reproducible in all hospitals and has reestablished the faith of the common man in government institutes.

## INTRODUCTION

India has rapidly grown over the past few decades from being a conservative society into a global, enterprising one. Indian plastic surgeons can take pride in the fact that India is fast becoming a major hub for cosmetic and medical tourism. In spite of that, there are still a large number of patients in Indian society who have treatable deformities, and yet remain untreated simply because they either don't have awareness of and/or access to medical facilities. In order to be a strong and self reliant country, this deformity load has to be decreased so that these socially inactive members of the community with correctable deformities can be brought back to the society to be productive citizens of the country.

Although exact statistics are not available, Indian plastic surgeons see around 7,00,000–8,00,000 burn admissions annually[1] with around 10,00,000 cleft patients yet to be operated.[2] In spite of this voluminous load, India does not have national health programs for the various deformities Indian plastic surgeons typically treat.

Project Muskan was initiated in our Department as an innovative model for targeting the above problems and is probably one of its kind in the field of plastic surgery in our country. It is unique because it is a perfect collaboration of government institutions, Non Government organization (NGOs), and cooperative organizations.

The government provided the infrastructure and the team of plastic surgeons while the raised funds and the milk dairy network helped in patient identification and awareness.

## METHODS

At the time of initiation of Project Muskan, the following realistic goals were set:

- To educate the population and create widespread awareness regarding correctable deformities.
- To provide means of transport, hospital stay facilities for the patients and their attendants absolutely free of cost.
- To provide high-quality health care to the patients by improving the technology and infrastructure of the department.
- To introduce similar models in other hospitals in the country.

Creating awareness was the most important goal for Project Muskan and was done in various villages by conducting awareness camps with the help of the nongovernment organization. At these camps, we took the opportunity to train Primary Health Centre (PHC) doctors and their staff as well as the leprosy workers in the PHC. We showed them presentations covering the spectrum of plastic surgery and the deformities that could be easily corrected so that they could help us in patient identification and awareness. Leprosy workers were included in the training as they usually come in contact with the lower socioeconomic strata of the society and thus, can easily help in patient identification.

**Figure 1:**
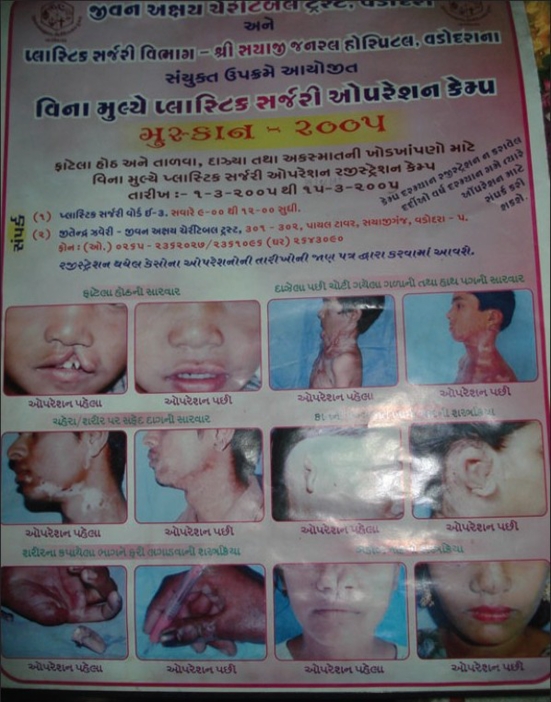
Poster in local language for awareness creation

**Figure 2:**
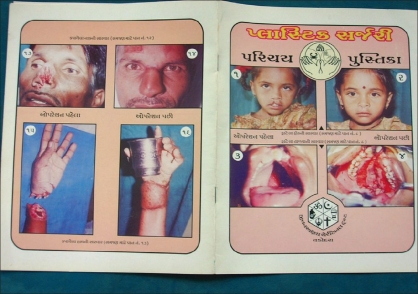
Booklet with short stories and photographs

Posters and pamphlets were created in the local language with preoperative and postoperative photos of patients and were distributed at the Panchayat (village level elected council) offices, schools and dairy offices [Figure 1]. Booklets were created with short stories and photographs of patients describing the vast improvement in the quality of life after surgery [Figure 2]. These booklets were also distributed during festive seasons such as *Navratri (An important festival in India and particularly in the state of Gujarat)* to further increase awareness. Due to the increased awareness, we started getting patients from villages and tribal areas.

The next step was to develop a working system. Village communities are closed and conservative societies with an illiterate and relatively poor population with numerous social taboos and superstitions. Villagers are still wary about cities and the complexities involved with them. They remain hesitant to come to government hospitals out of the simple fear of getting lost in the mad morning rush usually seen in outpatient departments. Hence, we realized the need of a system where these rural patients could be identified, selected, brought directly to us, treated, and finally taken back to their home when the treatment was over.

**Figure 3:**
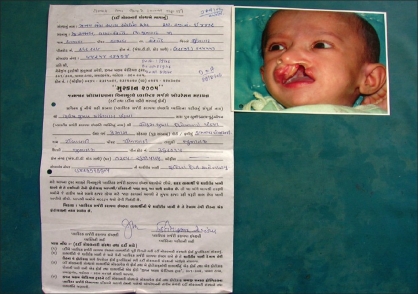
Application form with photograph of the patient

It is here that the nongovernmental organization, the Jeevan Akshay Charitable trust, became an indispensable member of the team. When a dairy worker went to a village for milk collection, he was requested to get a simple form filled out with a photo of the deformed patient [Figure 3]. These forms were collected and sent to the NGO office from where they were brought to our department. At the department, the forms were assessed and appointment dates were given to the patients. Four or five patients from the same locality were grouped and given a common date for admission so that travel became both convenient and comfortable for the patients. On that particular date, an NGO worker accompanied them to the hospital OPD/Ward where they were examined by doctors and admitted immediately. After being admitted to the ward, all the necessary investigations were done and the patient was posted for surgery. Thus, by giving a date for surgery right after the examination of the forms, repeated visits to the hospital were avoided.

**Figure 4:**
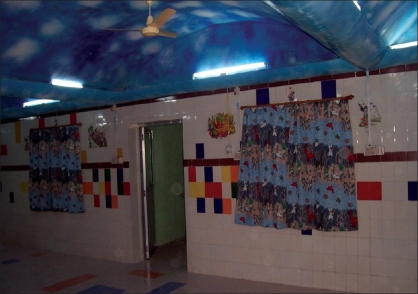
Renovated pediatric ward with cartoon murals and ceiling **with sky effect**

**Figure 5:**
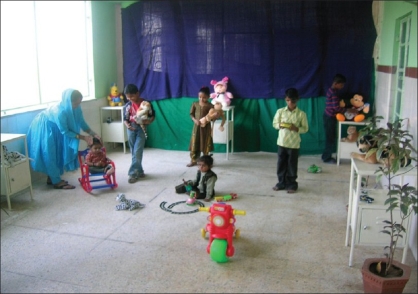
Separate playroom with toys for children

**Figure 6:**
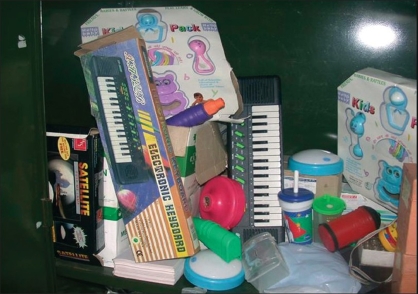
Toy bank in pediatric ward

The stay of the patient in the ward before the surgery was equally important. Patients' hospitalization presented a great opportunity for interaction with them and educating them about basic hygiene and cleanliness. As most of our patients were children, we were even more careful to decrease any hospitalization-related trauma or discomfort. With the aid of the NGO, the pediatric ward was renovated, its walls painted with cartoon murals, the ceilings made to look like the sky, and a separate play room assigned to house toys [Figures 4–6].

As our patients tended to come from very low socioeconomic strata of society, their untreated deformities were sometimes due to lack of awareness and sometimes due to poverty. To address this financial problem, the whole process was made absolutely free for the patients. The NGO paid for all the necessary awareness campaigns, transportation of patients with their attendants, investigations, medications, postoperative splints, and pressure garments including their expenses for follow-up visits. Hence, Project Muskan is a truly free comprehensive health care service provided at the doorstep of rural patients.

Once the treatment was over, the patients were discharged along with the necessary medications they needed and were taken back to their villages by NGO workers. Necessary pressure garments and splints were provided whenever required for individual patients, either at the time of discharge or during the subsequent follow-up visits, which were also taken care of by the nongovernmental organization.

Thus, the government, the non government organization, and the cooperative sector all had definite roles to play in the functioning of Project Muskan.

The role of nongovernment organizations included:

- Assistance through awareness-creating campaigns by printing posters, booklets, banners, and pamphlets.
- Traveling allowance given by NGO.
- Special investigations of patients that are not available in government institutes.
- Funding of any intra- and postoperative medications.
- Postoperative Splintage and pressure garments.
- Providing accommodation facilities for the patients' attendants.
- To appoint people who assisted the patients during their visit to the hospital.
- To manage screening and health camps in tribal areas and remote villages.
- To manage blood donation camps for assistance in the project.

The role of the Cooperative dairy:

- Providing application forms to patients through milk collection centers.
- Taking photographs of patients with their deformities to attach to the application forms.
- Collection of application forms and sending them to the NGO's office.
- Collection of forms from the NGO (with the date of admission) and sending them back to the patient.
- Provision of the vast network that penetrated into interior and the tribal areas.
- Tracking the patients in case they did not turn up on the respective dates and during follow-up.

The role of the Department and Government Hospital included providing:

- A team of plastic surgeons, anesthetists, and physiotherapists.
- A team of well trained nursing staff.
- Facilities for basic investigations and routine medications.
- Basic facilities for the operation theatre.

## RESULTS

*Benefits to Society:* Project Muskan currently has > 1500 registered patients and > 1000 patients have been operated in the past two years. All types of patients including those with clefts, hypospadius, microtia, and burn contractures have been operated upon under the auspices of this project. From a single district when it started in March 2005, Project Muskan now completely covers five districts (around 3000 villages and tribal areas) and many interior areas in western and northern Gujarat. Due to the widespread awareness and the facilities provided to the patients in this project, the message has spread beyond the state's boundaries and we are getting patients from neighboring states like Madhya Pradesh, Rajasthan, and Maharashtra. This brings the population covered by us to around 10,000,000. We have now started getting an increasing number of children with such deformities at stages when these can be corrected along with adult patients. We have also conducted various academic workshops at the department with national faculty coming to the department. Thus, the patients benefit from the technical skills and the experience of the faculty and interaction of our residents with this experienced faculty results in the improvement in training.

*Scientific advantages:* Most of the literature available for congenital deformities is from developed countries where surgeons see patients still in their early childhood, whereas in our country, the age of presentation is very late because of lack of awareness, illiteracy, and poverty of large sections of the population. The treatment protocol of western countries cannot be blindly applied for the management of these patients. Although we have a large number of patients in all government hospitals, we lack the ability to follow these patients after treatment, and so it becomes difficult to know the long-term results of our cases. However, because of project Muskan, follow-up has increased tremendously because the patients do not have to pay for the traveling charges. Case control studies for comparative analysis of different treatment protocols are now possible and we have already begun these investigations. Due to increased awareness, we have been able to treat an increasing number of patients with rare deformities. In the past one year, we have operated upon two cases of proboscis lateralis, a condition that is quite rare in that only about 50 such cases have been reported in the world literature. We also got the world's first case of complete cleft of the upper limb and are in the process of publishing our experience (see rare case report in this issue, Ed.).

*Benefits to the Department:* It is because of financial assistance from the NGO that a state-of-the-art operation theatre has been made available for Project Muskan. We have upgraded the infrastructure with an Image intensifier, drill systems, and other equipments using the donations.

Similarly the paediatric ward has been renovated into a play center with a toy bank with the help of which children can enjoy their stay in the hospital.

## DISCUSSION

Various projects and camps are being conducted in the country by various organizations but they mainly constitute operative camps over a short duration at regular intervals of time like those conducted by the Rotary and the Lions clubs. Some projects like the Smile Train Project address specific deformities such as clefts.[3] However, to the best of our knowledge, there is no ongoing program in the Plastic Surgery fraternity that helps to identify patients from the interior and tribal areas of the country and treats them free of cost, including the cost of travel. In the absence of a national health program for the patients we cater to as plastic surgeons, this Project works as an efficient substitute. The unique features of Project Muskan are as follows:

It is a perfect collaboration of the government sector, a nongovernmental organization, and the cooperative sector to provide comprehensive health care to the patients at their doorstep at no cost.

There is participation of the common man in creating awareness for identification of patients.

Involvement of dairy (milk cooperative sector) is unique to this project in utilizing their existing network of milk collection to reach tribal areas and villages. Their whole network is working for patient identification and awareness in these areas without adding to any additional expense to themselves or the government.

This pattern is easily reproducible, and can be initiated in hospitals having as few as 20 or as many as 2000 beds.

While in Gujarat, we took the help of the existing milk dairy network, this project can be modified according to the locally available network. In Punjab, the agroindustrial co-operatives can be used while in Maharashtra, assistance from the sugar cooperative could be taken. Other parallel networking systems such as the post offices and Aanganwadis could also be used.

As we are using a governmental set-up, this project reestablishes the faith of the common man in the existing health system.

We share the common dream of India becoming a developed nation by 2020. But with so many children and young adults with treatable deformities who remain untreated and hence, do not contribute to the development of the society due to social stigma, this dream will remain unrealized. By implementing these types of projects at various places in the country, using the available resources, we would be able to improve patient awareness and reduce the deformity load of the country. Thus, we could help to achieve the common goal of India becoming a developed country by 2020. Any corporate sector extending their support to these kinds of Projects should be encouraged. We recommend the initiation of such Projects in the country for the betterment of these large sections of impoverished and inacessible patients.

## References

[1] Ahuja R, Bhattacharya S (2004). Burns in the developing world and burn disasters. BMJ.

[2] Born with cleft lip? Get free surgery done here. - Article in the Times of India; 13th November 2007.

[3] Website of Smile Train foundation. http://www.SmileTrain.org.

